# On Temporal Patterns and Circulation of Influenza Virus Strains in Taiwan, 2008-2014: Implications of 2009 pH1N1 Pandemic

**DOI:** 10.1371/journal.pone.0154695

**Published:** 2016-05-03

**Authors:** Ying-Hen Hsieh, Hsiang-Min Huang, Yu-Ching Lan

**Affiliations:** 1 Department of Public Health, China Medical University, Taichung, Taiwan 40402; 2 Center for Infectious Disease Education and Research, China Medical University, Taichung, Taiwan 40402; 3 Department of Health Risk Management, China Medical University, Taichung, Taiwan 40402; Public Health Agency of Canada, CANADA

## Abstract

**Background:**

It has been observed that, historically, strains of pandemic influenza led to succeeding seasonal waves, albeit with decidedly different patterns. Recent studies suggest that the 2009 A(H1N1)pdm09 pandemic has had an impact on the circulation patterns of seasonal influenza strains in the post-pandemic years. In this work we aim to investigate this issue and also to compare the relative transmissibility of these waves of differing strains using Taiwan influenza surveillance data before, during and after the pandemic.

**Methods:**

We make use of the Taiwan Center for Disease Control and Prevention influenza surveillance data on laboratory-confirmed subtyping of samples and a mathematical model to determine the waves of circulating (and co-circulating) H1, H3 and B virus strains in Taiwan during 2008–2014; or namely, short before, during and after the 2009 pandemic. We further pinpoint the turning points and relative transmissibility of each wave, in order to ascertain whether any temporal pattern exists.

**Results/Findings:**

For two consecutive years following the 2009 pandemic, A(H1N1)pdm09 circulated in Taiwan (as in most of Northern Hemisphere), sometimes co-circulating with AH3. From the evolution point of view, A(H1N1)pdm09 and AH3 were able to sustain their circulation patterns to the end of 2010. In fact, A(H1N1)pdm09 virus circulated in six separate waves in Taiwan between summer of 2009 and spring of 2014. Since 2009, a wave of A(H1N1)pmd09 occurred every fall/winter influenza season during our study period except 2011–2012 season, when mainly influenza strain B circulated. In comparing transmissibility, while the estimated per capita weekly growth rates for cumulative case numbers (and the reproduction number) seem to be lower for most of the influenza B waves (0.06~0.26; range of 95% CIs: 0.05~0.32) when compared to those of influenza A, the wave of influenza B from week 8 to week 38 of 2010 immediately following the fall/winter wave of 2009 A(H1N1) pdm09 was substantially higher at r = 0.89 (95% CI: 0.49, 1.28), in fact highest among all the waves detected in this study. Moreover, when AH3 or A(H1N1)pdm09 exhibit high incidence, reported cases of subtype B decreases and vice versa. Further modeling analysis indicated that during the study period, Taiwan nearly experienced at least one wave of influenza epidemic of some strain every summer except in 2012.

**Discussion:**

Estimates of R for seasonal influenza are consistent with that of temperate and tropical-subtropical regions, while estimate of R for A(H1N1)pdm09 is comparatively less than countries in Europe and North America, but similar to that of tropical-subtropical regions. This offers indication of regional differences in transmissibility of influenza virus that exists only for pandemic influenza. Despite obvious limitations in the data used, this study, designed to qualitatively compare the temporal patterns and transmissibility of the waves of different strains, illustrates how influenza subtyping data can be utilized to explore the mechanism for various influenza strains to compete or to circulate, to possibly provide predictors of future trends in the evolution of influenza viruses of various subtypes, and perhaps more importantly, to be of use to future annual seasonal influenza vaccine design.

## Introduction

According to World Health Organization (WHO) statistics, influenza occurs globally with an estimated annual attack rate of 5%-10% in adults and 20%–30% in children [1]. These annual epidemics are estimated to result in approximately 3 to 5 million cases of severe illness worldwide and around 250 000 to 500 000 deaths, which is a major cause of losses in human lives and a grave global public health issue.

Seasonal influenza typically occurs with annual regularity in temperate regions in autumn and winter, mainly in the Northern Hemisphere from October to March and in the Southern Hemisphere from April to September. However, influenza cases are reported almost year-round in tropical regions. Taiwan is located in the tropical-subtropical region of the Northern Hemisphere, split north-south near the middle of the island by the Tropic of Cancer. Influenza cases are reported throughout the year with some summer influenza epidemics, but with peak number of reported cases occurring annually in autumn and winter from late October to March [2]. Moreover, studies have shown that excess influenza deaths during these months similar to those in the temperate zones can also observed in Taiwan [3–4]. It has also been proposed previously that Taiwan is an evolutionarily leading region for global circulation of influenza virus A (H3N2) [5]. Moreover, past studies on sequence comparison of seasonal influenza strains A(H1N1) in Taiwan versus vaccine strains have showed that many vaccine-like Taiwanese strains in 1995–2003 were circulating at least 2 years before the vaccine strains were introduced [6]. Hence investigating circulation patterns of seasonal influenza virus strains might shed lights on possible global circulation patterns in the following years.

There are 3 main types of circulating influenza viruses in the world: A, B, and C. Influenza viruses (of the family *Orthomyxoviridae*) are enveloped negative-strand RNA viruses with segmented genomes [7]. Of its two genera, one includes influenza A and B viruses, and the other influenza C virus. Type A influenza viruses are subtyped based on 16 known hemagglutinin (HA) and nine neuraminidase (NA) subtypes that exist in wild birds and provide a source of viral HA and NA subtypes antigenically novel to humans. The subtypes are further classified according to the combinations of virus surface proteins, abbreviated as H1–H16 and N1–N9 [8–9], starting with the 1918 swine H1N1 influenza which led to the 1918–1920 influenza pandemic. Its descendants include an H2N2 pandemic strain in 1957, which circulated until 1968 when it was replaced by H3N2 pandemic viruses. Among many subtypes of influenza A viruses (IAV), influenza A(H1N1) and A(H3N2) subtypes are currently circulating yearly among humans. Type C influenza cases occur much less frequently than that of A and B. Therefore, only influenza A and B viruses are included in seasonal influenza vaccines recommended every year by the WHO.

Curiously, pre-1957 strains of H1N1 strains reappeared in 1977, after which only H1N1 and H3N2 viruses have circulated globally in the form of seasonal epidemics, leading to some conjecture prior to 2009 that the next pandemic influenza will be caused by an H2 strain [10]. However, the 2009 A(H1N1)pdm09 pandemic took many by surprise, and demonstrated that the mechanism for circulation pattern of influenza strains is still poorly understood and it is practically impossible to predict the occurrence and the virus subtype of future pandemics.

Furthermore, pandemic influenza and seasonal influenza are undoubtedly linked, in ways also yet unascertained. Examining past influenza pandemics throughout history, it has been observed that strains of pandemic influenza also led to succeeding seasonal waves, albeit with decidedly different patterns. For example, while the 1889–1893 pandemic made three or more successive annual and largely seasonal reappearances, the 1918 pandemic spread in two or three rapidly recurring waves within a short period of time in many parts of the world (e.g., [11]) before settling into a pattern of annual seasonal recurrences [12]. Yet both of these pandemics resulted in high human mortality, especially that of 1918. On the other hand, the virus strains causing the 1957 (H2N2) and 1968 (H3N2) influenza pandemics, unlike their 1918 H1N1 virus predecessor, did not immediately produce rapidly successive waves of infections or multiple annual recurrences of high mortality, but settled more quickly into familiar patterns of annual seasonal endemic circulation [13–14].

In this work, we will focus on circulating influenza strains for influenza epidemics that have occurred in Taiwan shortly before and after the 2009 A(H1N1)pdm09 pandemic, namely between 2008–2014, by using Taiwan Center for Disease Control and Prevention (TCDC) Influenza Surveillance Data on laboratory-confirmed circulating strains [15]. The aim is to use mathematical modeling to pinpoint the temporal circulation and/or co-circulation of different strains in order to gauge the difference in their transmissibility, if any, and how they interact or compete for the human host population, with hope to provide some insights on how the 2009 pandemic impacts the circulation of seasonal influenza in the years following the pandemic.

## Methods and Materials

### Data

The laboratory confirmed influenza surveillance data used in this study was obtained from TCDC weekly Influenza Express, which is publicly available from TCDC website [15]. The data spans from week 1 of 2008 to week 18 of 2014, covering autumn/winter influenza seasons in Taiwan (data given in S1 Table). Nasal and/or throat swabs were collected from patients with influenza-like-illness (ILI) through 255 contracted healthcare facilities and laboratories in all parts of Taiwan (Table 1). Unavoidably, the number of samples taken varies greatly in time due to temporal changes in disease surveillance, in particular during a pandemic. For example, substantially larger number of samples was taken in the fall influenza season in 2009, and subsequently during similar seasons in 2010 and 2011, in an effort to ascertain the impact of A(H1N1)pdm09 infections in Taiwan inn post-pandemic years. These samples were tested for virus subtype with RT-PCR or virus isolation, and with HA or NA genes of the isolated virus sequenced. The samples were determined to be one of the five influenza virus strain: B, AH1, A(H1N1)pdm09, AH3, and H3N2, with A(H1N1)pdm09 isolated only after week 26 of 2009 and H3N2 only after week 33 of 2011.

**Table 1:** Number of laboratory confirmed influenza positive samples collected from patients with influenza-like-illness (ILI) from 255 TCDC-contracted healthcare facilities and laboratories in Taiwan.

Time Period (year/month)	# of samples tested	# of influenza positive sample	% of positive tests
2009/06-2010/06	16372	4174	25.5
2010/07-2011/05	13961	2948	21.1
2011/07-2012/06	15616	3310	21.2
2012/07-2013/06	8355	1129	13.5
2013/07-2014/05	7785	1342	17.2

(Source: Taiwan Center for Disease and Control, Taiwan Influenza Express)

### Richards model

In the Richards model [16], the weekly cumulative number of lab-confirmed cases at week t is denoted by *C(t)* and given by the following formula:

$$C(t)=K{\left[1+e^{-ra(t-t_{i}-(\text{ln}a)/ra)}\right]}^{-1/a}$$

Here *K* is the total number of cases over a wave of infections, *r* is the per capita growth rate of the cumulative case number, *a* is the exponent of deviation of the cumulative case curve, and *t*_*i*_ is the outbreak turning point of the cumulative epidemic wave, or the peak time of the outbreak incidence, signifying the moment of upturn or downturn for the temporal increase in cumulative case number. The Richards model has been applied extensively to model many infectious disease outbreaks [17–26].

When there is more than one wave of infections, as in our current study, a variation of the Richards model can be utilized, which makes the distinction between two types of turning points [23–27]; one type which occurs at the peak incidence, and a second type which occurs in a multi-wave epidemic when the growth rate of the cumulative case number begins to increase again, indicating the beginning of the next wave.

The basic reproduction number R_0_ for the Richards model is *R*_0_ = *exp*(*rT*), where *T* is the generation time or the average time period from onset of one individual to the onset of his/her contacts. Mathematically, given a growth rate *r*, this formula provides an upper bound for the basic reproduction number over all assumed distributions of the generation time T [28]. We note that in this study, we in fact obtain the effective reproduction number R, not the basic reproduction number R_0_, since all circulating influenza strains are descendants of some previously circulating virus and hence some level of pre-immunity in the population is already in place [29].

The model parameters of obvious epidemiological importance, namely, *K*, *r*, and *t*_*i*_, can be estimated for each wave of infections by fitting the weekly TCDC laboratory confirmed influenza strain surveillance data to the Richards model using any standard software with nonlinear least-squares (NLS) approximation tool, e.g., SAS, MATLAB, etc. (See S1 Text)

## Results

**Fig 1:**
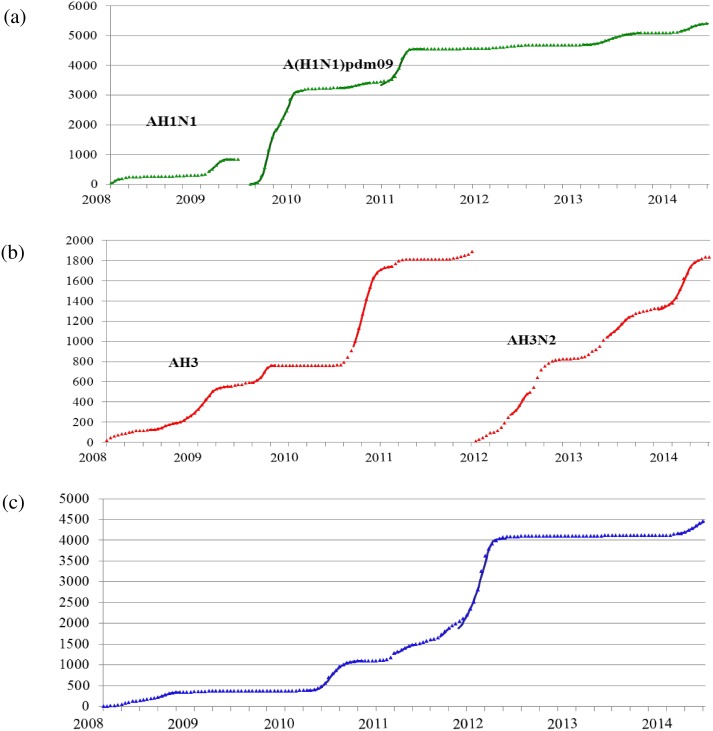
Model fits for the Richards model with cumulative influenza subtype data for strains. (a) AH1 and A(H1N1)pdm09; (b) AH3 and H3N2; (c) B.

We fit the weekly TCDC lab-confirmed case data by strain to the Richards model to pinpoint waves of circulating influenza strains in Taiwan during 2008–2012 (Fig 1). The list of waves that are found to be statistically significant for each set of the influenza subtype data, along with the estimated turning point and effective reproduction number R with 95% confidence intervals (CI) obtained for each wave, are given in chronological order in Table 2. For computation of the reproduction number, we use a generation time of T = 1.9/7 (95% CI: 1.30/7~2.71/7) which was obtained for the 2009 pH1N1 influenza pandemic [30] since the 2009 pH1N1 virus was found to be not significantly different from seasonal influenza in its transmissibility, although longer generation time has also been reported (see, e.g., [31]). Note that the 95% CIs for r and t_i_, taken from SAS output, are measures of uncertainty for the nonlinear least-squared estimation, while the 95% CI for R_0_ is computed, via the variances of r, also given in SAS output, and of T.

**Table 2:** Richards model fit for waves of circulating influenza strains in Taiwan, 2008–2014 in chronological order.

Time Period (year/week)	Strain	Growth Rate r (95% C.I.)	Turning point t_i_ (95% C.I.)	Reproduction number R (95% C.I.)
2008/01-06	AH1	0.77 (0.48,1.05)	1.8 (1.5,2.2)	1.23 (1.10,1.37)
2008/24-40	AH3	0.30 (0.22,0.38)	9.5 (8.8,10.2)	1.08 (1.04,1.12)
2008/31-42	B	0.06 (0.05,0.06)	4.9 (3.3,6.5)	1.02 (1.01,1.02)
2008/40-2009/16	AH3	0.10 (0.09,0.10)	14.7 (14.0,15.5)	1.03 (1.02,1.04)
2009/03-16	AH1	0.10 (0.08,0.13)	3.9 (2.3,5.5)	1.03 (1.02,1.04)
2009/26-40	A(H1N1) pdm09	0.59 (0.41,0.77)	9.4 (9.1,9.7)	1.17 (1.08,1.26)
2009/28-41	AH3	0.49 (0.38,0.60)	6.7 (6.4,7.1)	1.14 (1.08,1.21)
2009/40-2010/02	A(H1N1) pdm09	0.06 (0.05,0.06)	7.8 (6.1,9.4)	1.02 (1.01,1.02)
2010/08-38	B	0.89 (0.49,1.28)	12.0 (11.7,12.3)	1.27 (1.10,1.45)
2010/22-39	A(H1N1)pdm09	0.63 (0.34,0.92)	9.5 (9.2,9.8)	1.19 (1.07,1.31)
2010/31-52	AH3	0.15 (0.14,0.17)	5.0 (4.7,5.3)	1.04 (1.03,1.06)
2010/45-2011/16	A(H1N1) pdm09	0.18 (0.15,0.22)	11.9 (10.6,13.2)	1.05 (1.03,1.07)
2011/04-15	B	0.26 (0.19,0.32)	5.2 (4.6,5.8)	1.07 (1.04,1.11)
2011/29-34		0.08(0.08,0.09)	2.8 (2.6,3.0)	1.02 (1.01,1.03)
2011/39-2012/13		0.09 (0.08,0.09)	14.3 (12.7,16.0)	1.02 (1.01,1.03)
2012/13-24	AH3(H3N2)	0.31 (0.25,0.37)	5.9 (5.5,6.4)	1.09 (1.05,1.13)
2013/01-32	A(H1N1) pdm09	0.52 (0.27,0.77)	14.4 (13.9,14.8)	1.15 (1.05,1.25)
2013/14-28	H3N2	0.06 (0.06,0.06)	9.3 (7.9,10.6)	1.02 (1.01,1.02)
2013/36-2014/13		0.21 (0.17,0.26)	14.0 (13.2,14.9)	1.06 (1.03,1.09)
2014/02-18	A(H1N1) pdm09	0.53 (0.29,0.78)	5.9 (5.4,6.4)	1.16 (1.06,1.25)
2014/03-18	B	0.36 (0.25,0.47)	10.3 (9.9,10.7)	1.10 (1.05,1.15)

**Fig 2:**
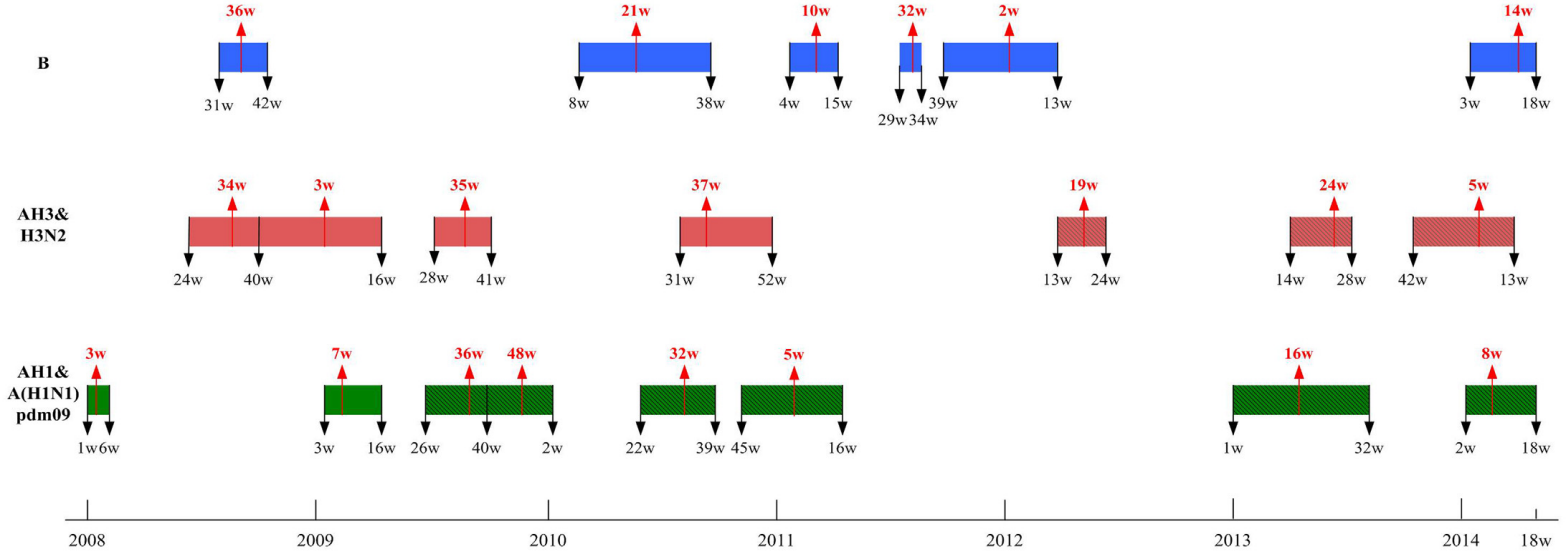
Timelines of waves of circulating influenza strains in Taiwan, 2008–2014. Blue for influenza virus strain B; red for AH3 and red shaded for H3N2; green for AH1 and shaded green for A(H1N1)pdm09.

**Fig 3:**
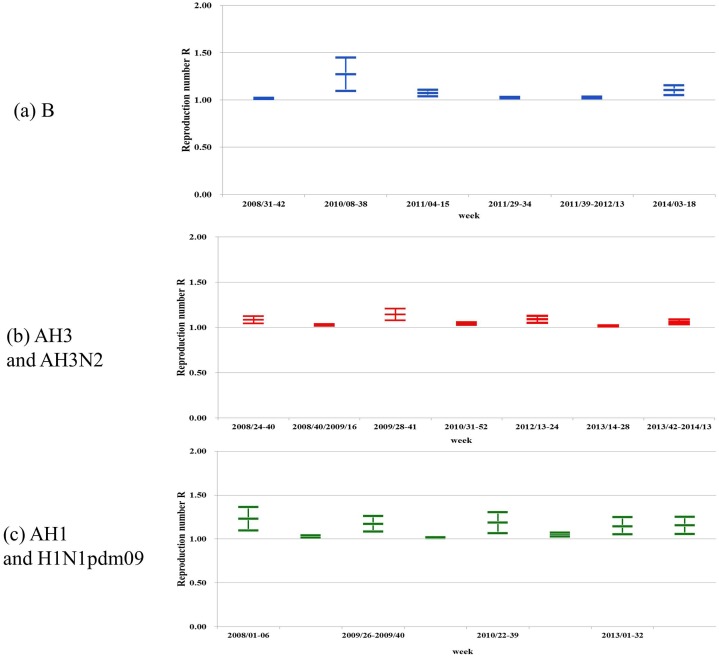
Reproduction numbers with 95% CI of circulating influenza strain waves. (a) B; (b) AH1; and (c) AH3, during 2008–2014 in Taiwan.

**Fig 4:**
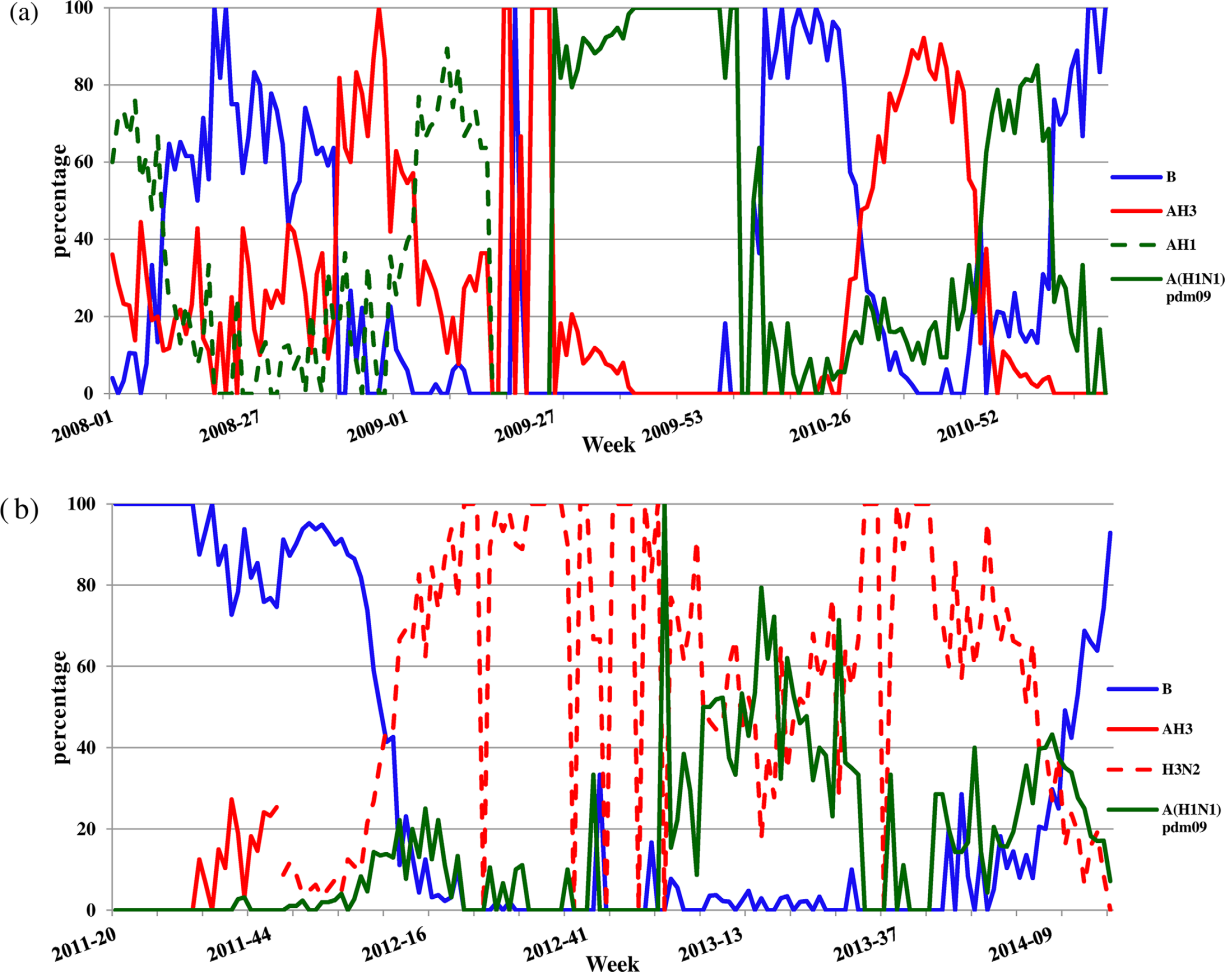
Weekly percentage of influenza strains among all lab-confirmed positive tests reported to Taiwan CDC by the contracted laboratories. (a) week 1, 2008-week 18, 2011; (b) week 20, 2011-week 18; 2014, with virus strains B in blue, AH1 in broken green, A(H1N1)pdm09 in green, AH3 in red, and H3N2 in broken red.

To further elucidate the temporally varying nature of the waves of circulating influenza strains, we illustrate the timeline of the waves in Fig 2. For clarity, we group the waves into three groups: B, H3/AH3N2, and AH1/A(H1N1)pdm09. To illustrate the temporal changes in transmissibility of the strains, we provide the reproduction numbers of circulating influenza strains in timelines (Fig 3). Fig 4 provides the percentage of each influenza strain among all lab-confirmed positive tests reported to Taiwan CDC by the contracted laboratories each week, which brings to light the dominating strain(s) during any time period.

## Discussion

Based on our results, we make the following observations:

### On temporal circulation patterns

1. It is known that, being located in tropical-subtropical region, Taiwan often experiences summer influenza epidemics. This is confirmed by our modeling, with a wave of some influenza strain almost every year during June-August, or around weeks 24–36 of each year, for example, AH3 in 2008, and A(H1N1)pdm09 in 2009 and again in 2010 (Fig 2).
2. A(H1N1)pdm09 virus circulated in six separate waves in Taiwan between summer of 2009 and spring of 2014 (Fig 2), although some of these waves were co-circulating with other strains. For example, the summer wave of 2010 (weeks 22–39) co-circulated first with B and later with AH3 (Fig 4a), while the two waves in 2013–2014 co-circulated with two respective waves of H3N2 (Fig 4b).
3. A(H1N1)pdm09 first appeared in Taiwan in two pairs of swiftly succeeding waves in less than two years, first in summer and autumn/winter of 2009, then again in summer and autumn/winter of 2010 (Fig 1a). It then occurred only sparingly for more than one year until another two waves, respectively at the beginning of 2013 and 2014, or the latter half of influenza seasons in Taiwan (Figs 1b and 2). The pattern is similar to what occurred in the aftermath of 1918–1919 pandemic [12]. Moreover, since 2009 a wave of A(H1N1)pmd09 occurred every fall/winter influenza season during our study period except 2011–2012 season, when influenza B circulated. We note that, although it did not return in the 2014–2015 influenza season, AH1N1 circulated again in Taiwan in the 2015–2016 season [32].
4. At most of the times during the time period under investigation, one of the three strains always tend to be the dominant circulating strain, even when co-circulation with some other strains occurred (Fig 4). One exception is the first half of 2013 (Fig 4b), where one would be difficult to conclude which, among A(H1N1)pdm09 and H3N2, is the dominant strain in that particular time period. Moreover, we are able to fit a wave of infections for *both* of these two strains during some parts of that half year (see Table 2 and Fig 1(a) and 1(b)). Although we note that a much larger study period than our data allows would likely offer more insights.
5. It has been proposed that, due to school/business closure due to traditional lunar New Year holidays in Taiwan that falls typically between week 4 to week 8 of each year, more frequent contacts in the households among family members who spent more time at home during the holidays tends to result in increased transmission [28]. It is interesting to note that during 2008–2014, at least one wave of cases was detected in the first two months of every year, including two circulating waves of B and A(H1N1)pdm09 in 2014 (Fig 2).

### On transmissibility of circulating strains

1. Interestingly, while the estimated per capita weekly growth rates of cumulative case number seem to be lower for most of the influenza B waves (0.06~0.26; range of 95% CIs: 0.05~0.32) when compared to those of influenza A, the wave of influenza B from week 8 to week 38 of 2010 immediately following the fall/winter wave of 2009 A(H1N1) pdm09 was substantially higher (at 0.89; 95% CI: 0.49, 1.28), in fact highest among all the waves detected in this study.
2. Although the A(H1N1)pdm09 virus caused significant increase in prevalence in Taiwan in 2009–2010 and for several years after, its transmissibility, as quantified by its reproduction number R, was relatively low when compared with past pandemics and actually not significantly greater than seasonal influenza virus strains in other years [33] (see Table 2). This observation corroborates previous studies reported in literature on the 2009 pH1N1 pandemic in other countries (e.g., [25, 30, 34–35]).
3. Most of the waves yielded reproduction number R between 1.0–1.2. Only two waves resulted in R great than 1.2; AH1 during weeks 1–6 2008 and B during weeks 8–38 2010 (Table 2 and Fig 3). Both waves exhibit wider 95% CI range, indicating greater uncertainty in model fit. We note that the former wave (AH1 in 2008) was in fact part of a wave that started in late 2007 (Fig 1a) which likely affected the fitting, and the latter wave (B in 2010) was occurring in the summer after the 2009 pH1N1 pandemic and co-circulating with a smaller wave of A(H1N1)pdm09 (Fig 2). The three waves with R between 1.1–1.2 occurred during and shortly after the 2009 pH1N1 pandemic, all co-circulated with other strain, and also exhibited wide 95% CI range.
4. Co-circulation of AH3 viruses with other strains is also of interest, in particular the circulation of AH3 virus almost in synchrony during the first (summer) wave of A(H1N1)pdm0 in July-September of 2009 (weeks 28–41, see Table 2), and two waves of H3N2 from 2013 to early 2014 that co-circulated with both B and A(H1N1)pdm09.
5. Another event that might have affected the transmissibility we observed was the mass immunization program initiated in Taiwan in late 2009 where more than five million of the 23 million Taiwanese were immunized with AdimFlu-S (unadjuvanted H1N1v from Adimmune) or Focetria^®^ (MF59^®^ adjuvanted H1N1v from Novartis) within five months [36–37]. Perhaps also indicative of the impact of herd immunity, the transmissibility of 2009 autumn/winter wave of pH1N1 was lower than that of the previous summer wave. Similarly in 2010, when R for the autumn/winter wave (when mass immunization program was again in place) was again lower than that of earlier summer wave. It is also interesting to note that the summer waves in 2009 and 2010 had almost identical transmissibility (R = 1.17 with very similar 95% CI ranges, Table 2) while R for autumn/winter waves in 2009 and 2010 were also comparable (1.02 in 2009 and 1.05 in 2010).

The estimates for R are consistently within range of other estimates for seasonal influenza in tropical-subtropical (e.g., Taiwan [29] and Australia [33]) and temperate regions (e.g., the United States and France [33]). However, the estimate of R during 2009 pandemic period is lower than that of many studies from other countries (see, e.g., [38]), but it is more similar to estimates obtained for Southern Hemisphere countries (Argentina, Brazil, Chile, Bolivia, Australia, New Zealand, and South Africa) [25, 35], as well as for Taiwan [34] and for Hong Kong [39]. All of these countries and territory have most of its territory in the tropical-subtropical region, which might be an indication of regional differences in transmissibility of influenza virus that exists only for pandemic influenza.

A study found that the hemagglutinin (HA) genetic and antigenic relatedness between H3N2 viruses circulating at the end of 2010 in Ontario, Canada, and A/Perth/16/2009 was likely the H3N2 component of the 2010–2011 [40]. This co-circulation of variants distinguished by specific AA substitutions was witnessed among H3N2 strains observed in Ontario, which are found to be related to the Victoria clade by using phylogenetic analysis. Due to the emergence of the Victoria 208 clade of A/Perth/16/2009 strain, WHO recommended a change to the H3N2 strain component of the 2012–2013 influenza vaccine, to A/Victoria/361/2011-like virus [41]. This highlights the hypothesis that antigenic drift among one subtype could still lead to epidemic event in human history. In this study, we present quantitative evidence of different values of R for different epidemic waves of the same influenza subtype (Fig 3). Recent studies have shown that antigenic drift might help to explain seasonal influenza circulation patterns in particular subtypes [42].

The study contains several obvious limitations, the foremost being the scarcity and incompleteness of full virology data. The data used came from TCDC influenza surveillance system data, where the samples were collected from patients with ILI from 255 contracted healthcare facilities and laboratories in Taiwan, which is not a random sampling of influenza cases in Taiwan. Unfortunately, the proportion of ILI cases tested is not known, and the proportion of positive tests varies from year to year (see Table 1). Moreover, the subtype-specific proportion of positive tests is not available from Taiwan CDC database. Hence we are unable to suitably adjust for the differences in the sampling rate, which results in unavoidable bias. This common but severe limitation in virology data size leads to uncertainty in our result. Since the proportion of asymptomatic cases might also be affected by specific subtype, this issue can only be satisfactorily resolved with comprehensive serotesting on the population level, which is not feasible given the limitation in laboratory testing today.

This data issue is even more glaring during summers, when there are typically less clinical cases and hence less number of samples being collected for laboratory testing. During the second half of 2012, no significant wave can be detected via the Richards model. However, we note that most of the samples tested during that time was determined to be H3N2 (Fig 4b), although the typically low weekly number of samples being tested during summer, due to more mild and subclinical cases in summers, became an obstacle for good model fitting. Nevertheless, the study, designed to qualitatively compare the temporal patterns and transmissibility of the waves of different strains, illustrates how influenza subtyping data can be utilized to explore the mechanism for various influenza strains to compete or to circulate, despite obvious data limitations.

Another limitation pertains to model fitting using cumulative data, which tends to have the advantage of smoothing out some of the stochastic variations which are often contained in longitudinal disease surveillance data [26]. Fitting cumulative data also has the potential drawback of introducing auto-correlation, potentially leading to biased high estimates of R [43]. Moreover, model fitting of cumulative disease data could also lead to errors in parameter estimates and subsequently in the corresponding CIs [44]. However, our interpretation of transmissibility deals mainly with the quantitative analysis on comparative variations in R for different strains and genotype variants using the same modeling framework, and hence is still valid.

In summary, our modeling study, using available government influenza subtyping data from routine influenza surveillance system, yields qualitative results on comparing circulation of different influenza strains which may shed some lights on the possible interaction between circulating influenza strains, in particular during and after a pandemic. There are many questions regarding the temporal patterns of circulation of influenza strains, in particular during and after a pandemic, and the 2009 pandemic offers us a great opportunity to learn, even with these hard to ignore biases in the subtype data. More importantly, it demonstrate the possibility that, with potential future advances in laboratory subtyping techniques that would enable us to be more easily accessible to more comprehensive information on the circulating influenza strains in a population, one can more readily ascertain the relationship, if any, between the evolution of influenza strains quantitatively such as we do in this study.

## Supporting Information

S1 Table (XLSX)

S1 Text (DOCX)
