# Supplementary material for: On Temporal Patterns and Circulation of Influenza Virus Strains in Taiwan, 2008-2014: Implications of 2009 pH1N1 Pandemic
Source: PLoS One. 2016 May 3;11(5):e0154695. doi: 10.1371/journal.pone.0154695 (PMC4854472; doi:10.1371/journal.pone.0154695)
Supplement: S1 Text — (DOCX) [file pone.0154695.s002.docx]

**File S2: Nonlinear least-squares (NLS) approximation tool: NLIN code in SAS**

DATA LG1;

INPUT S T ;

CARDS;

Data week

(case number) (week number)

. .

. .

;

data LG;

set LG1;

PROC NLIN DATA=LG METHOD=Gauss MAXITER=100 ;

PARMS R=0.1 K=1 to 30000 A=2 Ti=0 ;

bounds a>0;

MODEL S = K/(1+EXP(-R*A*(T-(Ti+log(A)/(R*A)))))**(1/A);

quit;
